# Efficacy and Safety of Transcatheter vs. Surgical Aortic Valve Replacement in Low-to-Intermediate-Risk Patients: A Meta-Analysis

**DOI:** 10.3389/fcvm.2020.590975

**Published:** 2020-11-16

**Authors:** Yake Lou, Yanan Gao, Ying Yu, Yanli Li, Ziwei Xi, Khaing Nyein Chan Swe, Yujie Zhou, Xiaomin Nie, Wei Liu

**Affiliations:** ^1^Department of Cardiology, Beijing Institute of Heart Lung and Blood Vessel Disease, Beijing Anzhen Hospital, Capital Medical University, Beijing, China; ^2^Department of Neurology, Beijing Tiantan Hospital, Capital Medical University, Beijing, China; ^3^Department of Geriatrics, The Affiliated People's Hospital of Inner Mongolia Medical University, Huhhot, China; ^4^International School of Capital Medical University, Beijing, China

**Keywords:** transcatheter aortic-valve replacement, TAVR, surgical aortic valve replacement, SAVR, meta-analysis

## Abstract

**Background:** The efficacy and safety of transcatheter aortic-valve replacement (TAVR) vs. surgical aortic valve replacement (SAVR) for low- to intermediate-surgical risk patients remains uninvestigated.

**Objectives:** We aimed to investigate the efficacy and safety of transcatheter aortic-valve replacement (TAVR) vs. surgical aortic valve replacement (SAVR) for low-intermediate surgical risk patients.

**Methods:** PubMed, Cochrane Library, and Embase databases were searched to identify potential references. Only randomized controlled trials (RCTs) or observational studies using propensity score matching were eligible for screening. The primary endpoint was all-cause death. The secondary outcomes were bleeding, stroke, myocardial infarction (MI), and other complications of aortic-valve replacement. In addition, we performed subgroup analysis based on surgical risk and study type.

**Results:** Eight RCTs and 13 observational studies covering 12,467 patients were included in the current meta-analysis. For patients with low-surgical risk, compared with SAVR, TAVR was found to be associated with a lower mortality at a follow-up period of 1 year (odds ratio, OR: 0.66, 95% CI: [0.46, 0.96], *P* = 0.03). This benefit disappeared when the follow-up was extended to 2 years (OR: 0.89, 95% CI: [0.61, 1.30], *P* = 0.56). For patients with intermediate-surgical risk, TAVR showed to have similar mortality with SAVR regardless of follow-up period (30-day, 1-year, or 2-year). TAVR could reduce the incidence of bleeding, AF, and AKI. For complications, such as MI and stroke, TAVR exhibited to have similar safety with SAVR. However, TAVR was found to be associated with a higher incidence of reintervention, major vascular complication, paravalvular leak, and PPI.

**Conclusion:** For patients with a low-to-intermediate surgical risk, TAVR has at least an equivalent clinical effect to SAVR for 2 years after the procedure.

## Introduction

Severe aortic stenosis (AS) is associated with a high mortality rate (1), and the most effective treatment for severe AS is aortic valve replacement (AVR) (2). The 2017 Joint American College of Cardiology and American Heart Association guidelines for the management of patients with valvular heart disease recommend surgical AVR (SAVR) as the first-line AVR method (2). Another alternative is transcatheter AVR (TAVR), which is a novel treatment strategy comprising a minimally invasive procedure to replace a narrowed aortic valve that fails to open properly (aortic valve stenosis) (2, 3). TAVR and SAVR share the same efficacy and safety in high-surgical-risk patients (2, 4, 5). However, although TAVR is not recommended as the optimal treatment in low- and intermediate-surgical-risk patients (2), ~90% of patients requiring AVR are considered low and intermediate surgical risk (6). Additionally, the prevalence of TAVR is increasing in low- and intermediate-surgical-risk patients (7). Hence, it is essential to investigate the efficacy and safety of TAVR vs. SAVR in patients with low-to-intermediate surgical risk.

## Materials and Methods

This meta-analysis was conducted in accordance with the Preferred Reporting Items for Systematic Reviews and Meta-Analyses (PRISMA) guidelines and the recommendations of the Cochrane Collaboration and Meta-analysis of Observational Studies in Epidemiology (8, 9).

### Search Strategy

The PubMed, Cochrane Library, and Embase databases were searched from inception until February 28, 2020, to identify potentially relevant references using MESH words and keywords (title/abstract). We also performed a manual search of relevant references. All studies comparing TAVR with SAVR, including randomized controlled trials (RCTs) and observational studies, were identified using the filters presented in Harvard Countway Library. The search details are listed in the Supplementary Materials.

### Inclusion Criteria

1. RCTs or observational studies using propensity score matching (PSM) that compared TAVR and SAVR in patients classified as having a low or intermediate surgical risk.
2. Risk stratification reported in the article.

### Exclusion Criteria

1. Minimally invasive SAVR.
2. Patients with a history of failed surgical aortic bioprosthesis implantation.
3. For observational studies, the Society of Thoracic Surgeons (STS) score or European System for Cardiac Operative Risk Evaluation (EuroSCORE) significantly differed between the TAVR and SAVR groups after PSM.
4. Being a single-arm prospective study.

### Definitions of Low and Intermediate Surgical Risks

Low surgical risk was defined as a mean STS score of <4% and/or a logistic EuroSCORE of <10%. Intermediate surgical risk was defined as a mean STS score of 4–8% and/or a logistic EuroSCORE of 10–20%.

### Data Extraction

Two authors (Lou and Yu) independently screened eligible studies and evaluated the study quality. Another two authors (Xi and Swe) independently extracted the baseline and outcome data. Disagreements were resolved by another two authors (Zhou and Li). Incomplete data were obtained by contacting the corresponding authors or browsing other published articles.

### Outcomes

The primary outcome was all-cause mortality. The secondary outcomes were bleeding, stroke, myocardial infarction (MI), atrial fibrillation (AF), reintervention, major vascular complication, paravalvular leak, permanent pacemaker implantation (PPI), and acute kidney injury (AKI). All outcomes were assessed at 30 days, 1 year, and 2 years after AVR.

### Risk of Bias

For RCTs, the risk of bias was evaluated in accordance with the Cochrane Handbook for Systematic Reviews of Interventions (version 5.1.0). For observational studies, the study quality was assessed using the Risk of Bias Assessment Tool for Non-randomized Studies (RoBANS) tool (10).

### Statistical Analysis

All statistical analyses were performed using Review Manager (RevMan) 5.3 (The Cochrane Collaboration, Copenhagen, Denmark) and Stata 15.1 (Stata Corp., College Station, TX, USA) software.

The odds ratio (OR) and 95% confidence interval (CI) were used to compare the clinical outcomes of TAVR vs. SAVR. The I^2^ statistic was used to test the heterogeneity across studies. The Mantel–Haenszel random-effects model was employed to calculate the OR and 95% CI given the possible heterogeneity across PSM studies and RCTs. In addition, subgroup analyses were carried out based on the data source (RCT or observational study) and surgical risk (low or intermediate). Sensitivity analysis was undertaken by the sequential exclusion of one trial. Publication bias was assessed using a visual funnel plot and Begg's test.

## Results

### Study Selection

**Figure 1:**
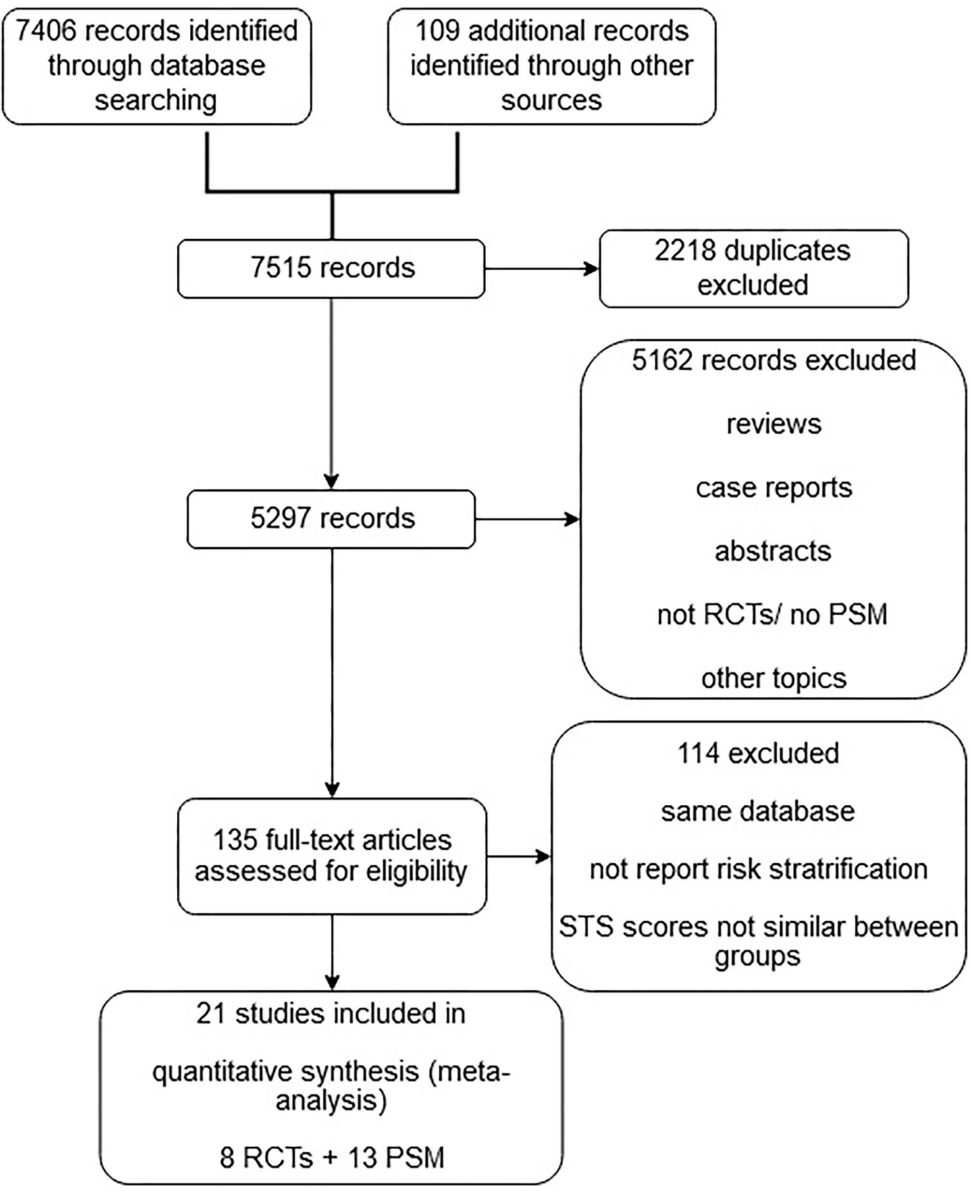
Flowchart.

The initial search identified 7,515 potentially relevant articles. A total of 2,218 duplicates, 847 reviews, 325 case reports, 986 abstracts, 854 non-RCTs, and 2,150 studies with nonrelevant topics were excluded. The full-text versions of the remaining 135 articles were assessed. A final total of 21 studies with 12,467 patients were eligible for meta-analysis after the exclusion of studies that did not report the risk stratification and/or had significant differences in STS scores between the TAVR and SAVR groups. Of the 21 eligible studies, eight were RCTs, and 13 were observational studies (Figure 1).

### Characteristics of Eligible Studies

Of the 12,467 patients, 6,329 (50.7%) received TAVR and 6,138 (49.3%) received SAVR. Furthermore, 4,123 of 6,329 (65.1%) patients who underwent TAVR were from RCTs and 3,932 of 6,138 (64.1%) patients who received SAVR were from RCTs. The number of patients in each study ranged from 60 to 2,032. Patients' baseline data are listed in Table 1.

**Table 1:** Baseline characteristics of included studies.

**Study ID**	**Study** ** design**	**Data source**	**Follow-up**	**Sample size** ** (TAVR/SAVR)**	**Age** ** (years)**	**DM**	**Hypertension**	**COPD**	**CAD**	**PCI**	**STS score**	**Valve for TAVR**	**AVG (mmHg)**	**AVA (cm^**2**^)**
Makkar, (2020) (11)	RCT	PARTNER 2	5 year	1011/1021	82/82	38/34	/	31.8/30.0	69/67	27/28	5.8/5.8	Edwards Lifesciences SAPIEN XT heart-valve system	45/45	0.7/0.7
Reardon, (2017) (12)	RCT	SURTAVIC	2 year	864/796	80/80	34/35	93/90	/	63/64	21/21	4.4/4.5	84% CoreValve bioprosthesis/16% Evolut R bioprosthesis	/	/
Thyregod et al. (2019) (13)	RCT	NOTION	5 year	145/135	79/79	18/21	71/76	12/12	/	8/9	2.9/3.1	Medtronic CoreValve System(TM)	/	/
Mack et al. (2019) (14)	RCT	PARTNER 3	2 year	496/454	73/74	31/30	/	5/6	28/28	/	1.9/1.9	SAPIEN 3 system	49/48	0.8/0.8
Popma, (2019) (15)	RCT	Evolut Low Risk Trial	1 year	725/678	74/73	31/31	85/83	15/18		14/13	1.9/1.9	3.6% CoreValve/74.1% Evolut R/22.3% Evolut PRO	47/47	0.8/0.8
Toff (2020) (16)	RCT	UK TAVI	1 year	458/455	81/81	23/25	72/72	/	30/32	12/9	2.6/2.7	SAPIEN/CoreValve/Evolut/others	/	0.7/0.7
Nielsen, (2012) (17)	RCT	STACCATO	1 year	34/36	80/82	3/8	/	3/3		/	3.1/3.4	Edwards Lifesciences SAPIEN	/	0.7/0.7
Gleason, (2018) (18)	RCT	US-CoreValve High Risk Study	5 year	390/357	83/83	35/45	95/96	/	75/76	34/38	7.3/7.5	CoreValve self-expanding prosthesis	/	/
Fusari, (2012) (19)	PSM	Italy, 2008–2009	1 year	30/30	81/78	23/7	93/77	30/33	37/33	/	6.6/6.1	/	53/52	0.7/0.7
Virtanen, (2019) (20)	PSM	FinnValve registry	3 year	304/304	78/78	22/22	/		19/19	17/16	2.1/2.1	/	/	/
Latib, (2012) (21)	PSM	Italy, 2003–2008	1 year	111/111	81/80	19/22	70/69	26/23	40/46	/	4.6/4.6	58.3% EdwardsSAPIEN, Edwards-SAPIEN XT/ 41.7% CoreValve		
Tamburino, (2015) (22)	PSM	OBSERVANT, 2010–2012, Italy	1 year	650/650	81/80	25/25	/	22/22	/	15/13	9.5/10.2 (LES)	SAPIEN/CoreValve	51/51	0.7/0.7
Schaefer, (2019) (23)	PSM	University Heart Center Hamburg, Hamburg, Germany	30 day	109/109	76/74	16/22	/	/	30/30	/	2.0/2.0 (LES 2)		44/42	0.8/0.8
Tzamalis et al. (2020) (24)	PSM	TAVI Karlsruhe registry	6 year	216/216	78/78	/	/	/	48/48	/	8.7/8.8 (LES)	SAPIEN/CoreValve	/	/
Castrodeza et al. (2016) (25)	PSM	Hospital Clínico Universitario de Valladolid, Valladolid, Spain, 2009–2014	1 year	70/70	79/78	37/26	64/73	30/16	/	/	4.6/4.3	SAPIEN/CoreValve	50/50	0.6/0.7
Auffret, (2017) (26)	PSM	Québec Heart and Lung Institute, Québec, Canada, and Rennes University Hospital, Rennes, France, 2007–2015	1 year	71/71	74/73	/	/	/	44/61	/	4.4/4.4	SAPIEN/CoreValve	44/37	/
Piazza et al. (2013) (27)	PSM	SURTAVI-PSM	1 year	255/255	81/80	31/24	87/81	19/16	62/61	/	17.3/17.6 (LES)	/	/	/
Osnabrugge, (2012) (28)	PSM	TAVR or SAVR at the Erasmus MC, Rotterdam, Netherlands	1 year	42/42	79/79	26/19	/	24/19	48/48	/	12.9/12.5 (LES)	/	/	/
Kawashima, (2017) (29)	PSM	OCEAN-TAVI registry	30 day	166/166	86/85	/	81/74	21/21	/	25/28	7.1/6.2	SAPIEN XT	46/51	0.6/0.6
Sponga, (2017) (30)	PSM	University Hospital of Udine, Italy	30 day	40/40	88/87	15/15	70/80	30/23	38/58	/	3.2/3.2 (EuroScore II)	SAPIEN/CoreValve	44/46	0.6/0.7
Repossini, (2017) (31)	PSM	7 European cardiac centers, 2010–2014	30 day	142/142	76/76	29/30	61/59	/	/	/	7.2/6.7	/	48/49	0.7/0.7

### Primary Endpoint

**Figure 2:**
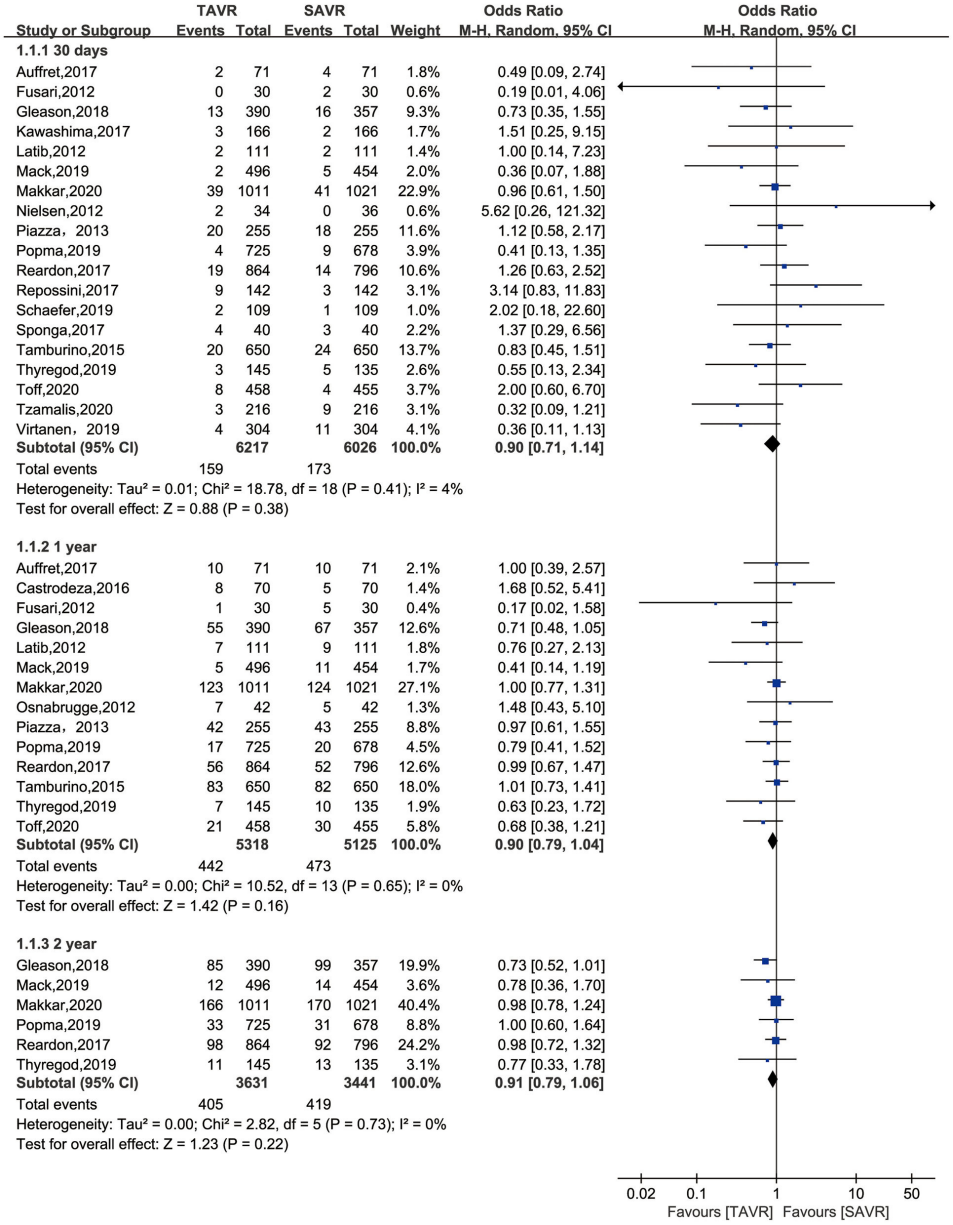
All-cause mortality of TAVR vs. SAVR in low-intermediate surgical risk patients at 30 days, 1 year, and 2 years.

**Figure 3:**
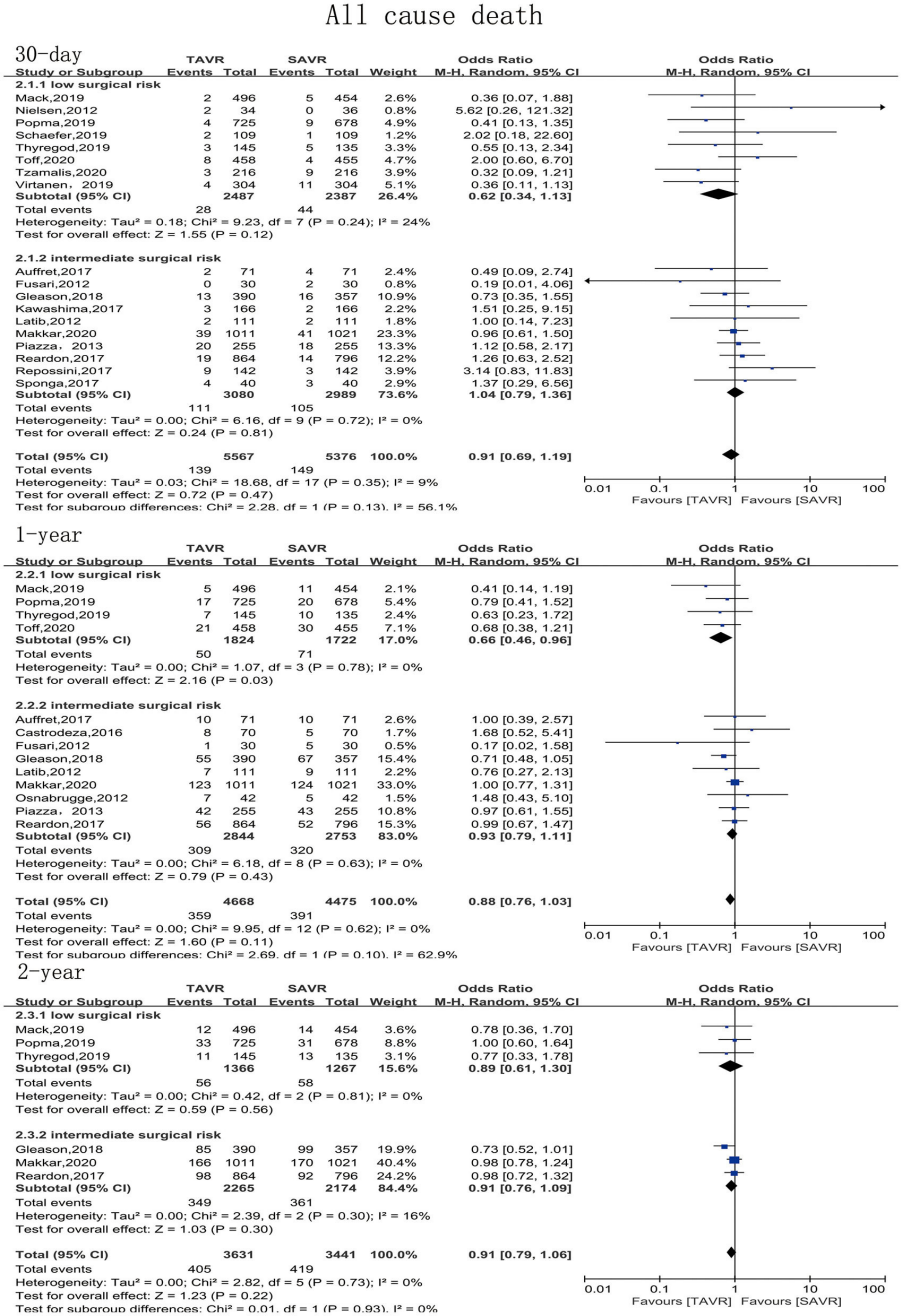
Subgroup analysis of all-cause mortality based on surgical risk stratification (low/intermediate).

**Figure 4:**
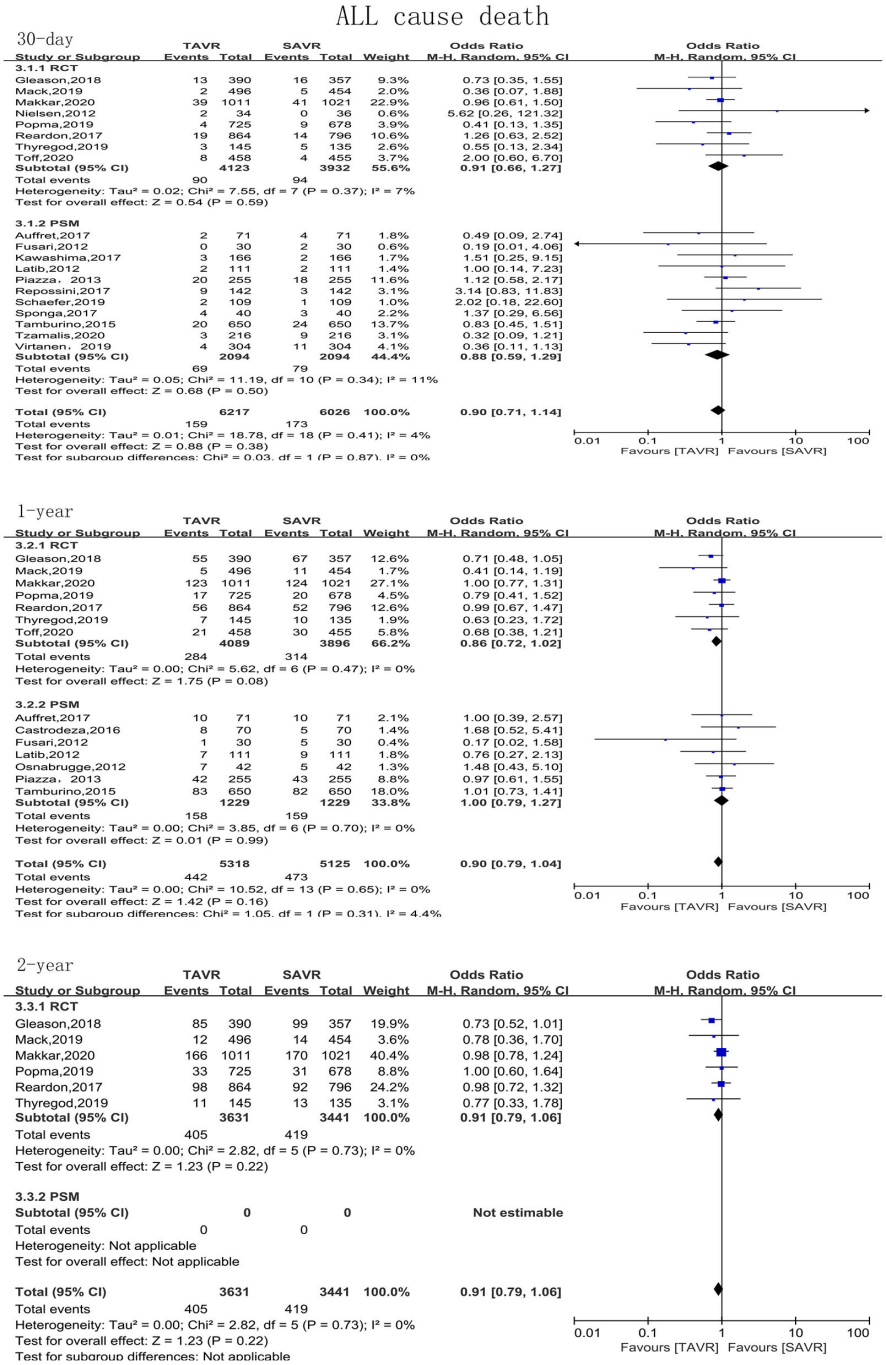
Subgroup analysis of all-cause mortality based on study type (RCTs/observational studies).

The primary endpoint of the present meta-analysis was all-cause mortality at 30 days, 1 year, and 2 years after AVR; the analysis of mortality at each of the timepoints was performed using data from 19, 14, and six studies, respectively. As shown in Figure 2, TAVR and SAVR provided similar clinical benefits at 30 days (OR: 0.90, 95% CI: [0.71, 1.14], *P* = 0.38), 1 year (OR: 0.90, 95% CI: [0.79, 1.04], *P* = 0.16), and 2 years (OR: 0.91, 95% CI: [0.79, 1.06], *P* = 0.22). Subgroup analysis showed that low-risk patients undergoing TAVR had a lower 1-year mortality rate than those undergoing SAVR (OR: 0.66, 95% CI: [0.46, 0.96], *P* = 0.03), while this advantage disappeared when the follow-up was extended to 2 years (OR: 0.89, 95% CI: [0.61, 1.30], *P* = 0.56). For intermediate-risk patients, the mortality rate did not significantly differ between patients undergoing TAVR and SAVR at any timepoint (30-day mortality (OR: 1.04, 95% CI: [0.79, 1.36], *P* = 0.81); 1-year mortality (OR: 0.93, 95% CI: [0.79, 1.11], *P* = 0.43); and 2-year mortality (OR: 0.91, 95% CI: [0.79, 1.06], *P* = 0.30) (Figure 3). The subgroup analysis based on data source did not find any significant difference between RCTs and PSM studies (Figure 4).

### Secondary Endpoints

**Figure 5:**
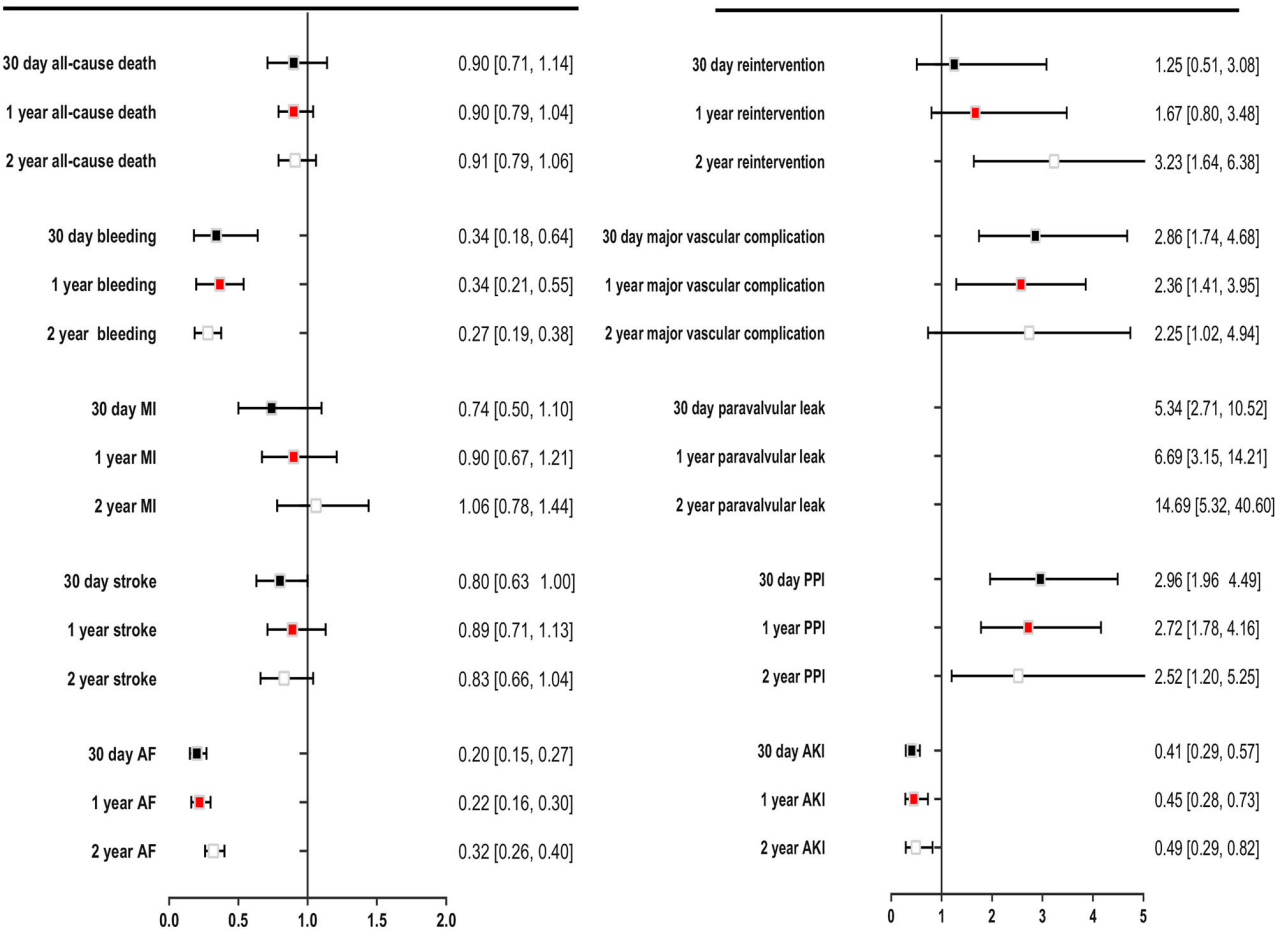
Comparing overall safety and efficacy outcomes for TAVR with SAVR for low- to intermediate surgical risk patients.

The secondary endpoints were bleeding, MI, stroke, AF, reintervention, major vascular complication, paravalvular leak, PPI, and AKI. TAVR significantly reduced bleeding events compared with SAVR during 30 days of follow-up (OR: 0.34, 95% CI: [0.18, 0.64], *P* = 0.001), even if the follow-up was extended to 1 and 2 years. TAVR was associated with lower incidences of AF and AKI than SAVR at each of the follow-up timepoints. Compared with SAVR, TAVR reduced the incidences of AF and AKI by 51 and 80%, respectively. The incidences of MI and stroke were similar in patients undergoing TAVR and SAVR. The 2-year postoperative incidences of stroke in the TAVR and SAVR groups were 6.8 and 8.1% (*P* = 0.09), respectively, and the incidence of MI was low in both groups, at approximately 3.0%. Compared with SAVR, TAVR increased the risks of reintervention, major vascular complication, paravalvular leak, and PPI at each of the follow-up timepoints; the corresponding OR and 95% CI for these variables were 3.23 [1.64, 6.38], 2.25 [1.02, 4.94], 14.69 [5.32, 40.60], and 2.52 [1.20, 5.25], respectively (Figure 5).

### Publication Bias and Quality Assessment

No substantial publication bias was noted in the funnel plot. The risk of bias in RCTs was mainly related to blinding, which could not be avoided. The detailed study quality assessment results are listed in the Supplementary Materials.

### Sensitivity Analysis

A sensitivity analysis performed after the exclusion of one study revealed that our results were robust.

## Discussion

To the best of our knowledge, the present meta-analysis is the largest one to compare the efficacy and safety of TAVR vs. SAVR in the low-to-intermediate-surgical-risk population. For patients with a low surgical risk, TAVR was associated with a lower mortality rate within 1 year than SAVR; however, this positive influence disappeared when the follow-up period was extended to 2 years. For patients with an intermediate surgical risk, SAVR and TAVR resulted in similar mortality rates. Compared with SAVR, TAVR reduced the incidences of bleeding, AF, and AKI but increased the incidences of reintervention, major vascular complication, paravalvular leak, and PPI. The incidences of MI and stroke were similar after TAVR and SAVR.

At present, observational studies show that patients with an intermediate surgical risk have a similar mortality rate after either TAVR or SAVR (25, 27). However, as this result is based on studies with small sample sizes and few RCTs, it is hard to definitively conclude that TAVR is as safe as SAVR. Our meta-analysis included three RCTs and nine observational studies and confirmed the safety of TAVR in patients with a low-to-intermediate surgical risk.

For patients classified as having a low surgical risk, it remains controversial whether TAVR benefits patients more than SAVR. Vipparthy and Levett found that TAVR reduces the mortality rate of low-surgical-risk patients compared with SAVR (32, 33), while Witberg et al. drew the opposite conclusion that low-surgical-risk patients have a higher mortality rate after TAVR than SAVR (34). The most recent RCTs showed that the 1-year mortality rate does not significantly differ between patients undergoing TAVR vs. SAVR (14, 16). Our subgroup analysis of eight studies found that TAVR was associated with a lower mortality rate in the first year of follow-up, but this benefit disappeared when the follow-up period was extended to 2 years. Witberg et al. drew their conclusion by analyzing data from four studies with different follow-up periods, ranging from several weeks to 3 years. In contrast, we synthesized our data using studies with same follow-up periods, which may cause less bias; furthermore, the sample size of our meta-analysis is almost twice that in the study by Witberg et al. These factors may suggest that our conclusion is more reliable than that of Witberg et al.

For patients with a low surgical risk, TAVR was superior to SAVR during a follow-up period of 30 days or 1 year, whereas the benefit disappeared when the follow-up was extended to 2 years. Another study found that TAVR and SAVR result in similar mortality rates during 30 days of follow-up, but TAVR results in a much lower 3-year survival rate than SAVR (35). As low-risk patients may be younger than patients with a higher surgical risk, they have a longer anticipated life. Thus, the long-term efficacy of TAVR should be evaluated in low-risk patients. Moreover, the long-term durability of SAVR needs to be evaluated during at least 10 years of follow-up (36). The longest follow-up period for TAVR in low-risk patients was just 6 years (24), and the result of this 6-year follow-up indicated that TAVR is associated with a higher all-cause mortality rate. Only one RCT reported the 5-year outcome of TAVR vs. SAVR in low-surgical-risk patients (13); this RCT found that TAVR is as safe as SAVR but only had a small sample size of 145 in the TAVR group and 135 in the SAVR group. Although the present meta-analysis included six studies with 7,072 patients, it was still not powerful enough to demonstrate the safety and efficacy of TAVR vs. SAVR. Furthermore, the relatively short length of the follow-up in the included studies meant that the long-term efficacy of TAVR could not be evaluated. Thus, further studies are required to investigate the long-term efficacy of TAVR in low-to-intermediate-surgical-risk patients.

TAVR has evolved since its inception. Procedural safety and bioprosthetic valve performance have improved with the implementation of new-generation TAVR devices, advanced imaging planning, growing operator experience, and new delivery systems. TAVR valve systems have undergone major innovations since the first TAVR procedure was performed in 2002 (37). For example, the newest SAPIEN valve system (SAPIEN 3 Ultra, Edwards Lifesciences) is the third generation of SAPIEN valve system, following the SAPIEN, SAPIEN XT, and SAPIEN 3 (38). SAPIEN 3 Ultra has an improved delivery system compared with SAPIEN 3. The improvements in TAVR devices and delivery systems have contributed to the current success of TAVR. The procedural outcomes of TAVR are also influenced by factors such as the learning curve and advanced imaging planning. CT is currently used to assess vascular access and plays a primary role in TAVR planning (39). Furthermore, CT can be used to accurately assess the aortic root and provide a reliable measurement of the aortic annulus. These factors contribute to a lower procedural failure rate in TAVR. Part of the success of the recent PARTNER 3 study is due to the support of preprocedural CT imaging (14). These improvements may have caused TAVR to gain better clinical outcomes. More clinical trials are needed to evaluate the safety and efficacy of TAVR.

Almost all included studies found that TAVR increases the risk of PPI; thus, the current meta-analysis found that the incidence of PPI was 14.7% in the TAVR group vs. 5.6% in the SAVR group. The predictors of the need for PPI are old age, a thick interventricular septum, and a high logistic EuroSCORE (40). Patients must be assessed for the presence of these risk factors to enable the selection of the best AVR treatment strategy. The 2-year incidences of other complications, such as MI, stroke, reintervention, major vascular complication, paravalvular leak, and AKI, were <10%. However, the rates of bleeding and AF were extremely high in the SAVR group. The incidence of bleeding was 17.0% in the TAVR group vs. 44.3% in the SAVR group, which is consistent with the findings of the PARTNER-I study (41). Another common complication after AVR is AF, with an incidence of 18.3% in the TAVR group vs. 39.0% in the SAVR group. A previous study reported AF rates of about 35 and 60% in the TAVR and SAVR groups, respectively (42). This discrepancy regarding the AF rates in the present meta-analysis vs. the previous study may be attributed to advances in the TAVR device, as the TAVR device has progressed to the third generation. In summary, TAVR was not associated with a higher complication rate than SAVR.

## Limitations

The present meta-analysis has several limitations besides those inherent in the original studies. First, the meta-analysis included both RCTs and observational studies, which may have resulted in bias; however, the subgroup analysis found that the results were robust, regardless of study type. Second, the meta-analysis was based on a study level instead of a patient level, as the raw data were not available; this prevented further subgroup analyses based on baseline characteristics. Third, some studies had a sample size of <100, which may cause bias. Fourth, a number of eligible patients may have had a higher STS score than the mean STS score used in the meta-analysis, which might have resulted in an underestimation of the real effect of TAVR. Fifth, some included studies did not provide echocardiographic baseline characteristics and outcomes, and so the data could not be analyzed. Finally, owing to the absence of data, the safety of TAVR during a longer follow-up period could not be investigated, although a longer follow-up may be of great significance.

## Conclusions

For patients with a low-to-intermediate surgical risk, TAVR has at least an equivalent clinical effect to SAVR for up to 2 years after the procedure.

## Data Availability Statement

The original contributions presented in the study are included in the article/Supplementary Materials, further inquiries can be directed to the corresponding author/s.

## Author Contributions

WL and XN came up the idea and supported the work. YLo and YG write the manuscript. YLo and YY independently screened eligible studies and evaluated the quality of studies. ZX and KS independently extracted the baseline and outcome data. Disagreement was resolved by YZ and YLi. All authors contributed to the article and approved the submitted version.

## Conflict of Interest

The authors declare that the research was conducted in the absence of any commercial or financial relationships that could be construed as a potential conflict of interest.
