# Supplementary material for: Efficacy and Safety of Transcatheter vs. Surgical Aortic Valve Replacement in Low-to-Intermediate-Risk Patients: A Meta-Analysis
Source: Front Cardiovasc Med. 2020 Nov 16;7:590975. doi: 10.3389/fcvm.2020.590975 (PMC7701058; doi:10.3389/fcvm.2020.590975)

**Study bias-RCTs**


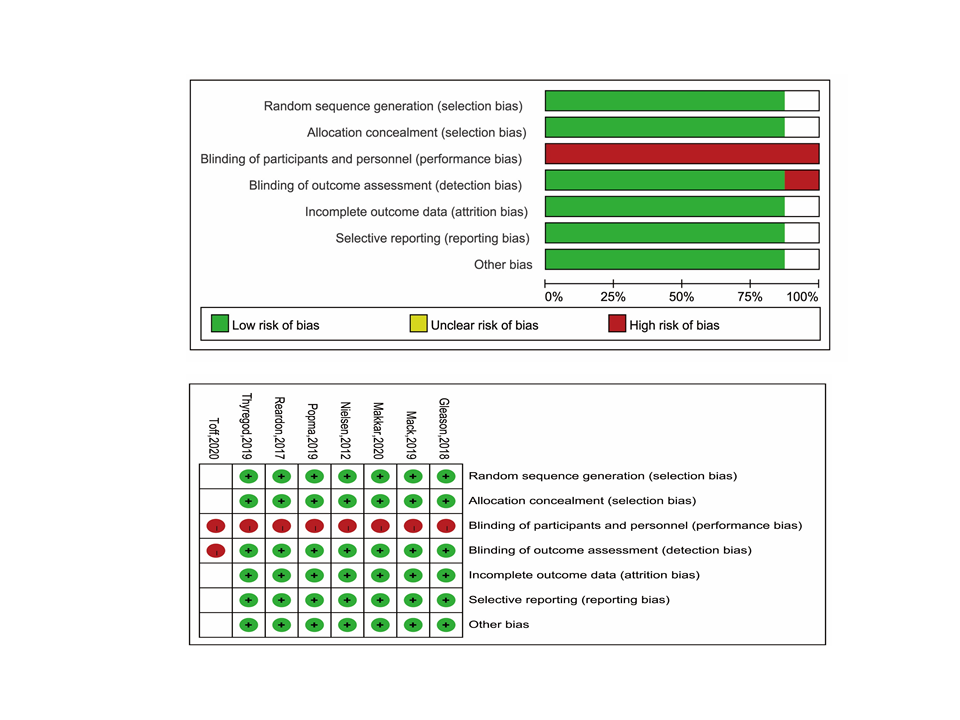


**Study bias-Propensity score-matched studies (Newcastle-Ottawa scale)**

| Study ID | Selection | | | | Comparability | Outcome | | | Overall |
| --- | --- | --- | --- | --- | --- | --- | --- | --- | --- |
|  | Representativeness of exposed cohort | Selection of non-exposed cohort | Ascertainment of exposure | Demonstration that outcome of interest was not present at start of study | Comparability of cohorts on the basis of the design or analysis | Assessment of outcome | Was follow-up long enough for outcomes to occur | Adequacy of follow-up of cohorts | Total score |
| Fusari,2012 | 1 | 1 | 1 | 1 | 2 | 1 | 1 | 1 | 9 |
| Virtanen，2019 | 1 | 1 | 1 | 1 | 2 | 1 | 1 | 1 | 9 |
| Latib,2012 | 1 | 1 | 1 | 1 | 2 | 1 | 1 | 1 | 9 |
| Tamburino,2015 | 1 | 1 | 1 | 1 | 2 | 1 | 1 | 1 | 9 |
| Schaefer,2019 | 1 | 1 | 1 | 1 | 2 | 1 | 0 | 1 | 8 |
| Tzamalis,2020 | 1 | 1 | 1 | 1 | 2 | 1 | 1 | 1 | 9 |
| Castrodeza,2016 | 1 | 1 | 1 | 1 | 2 | 1 | 1 | 1 | 9 |
| Auffret,2017 | 1 | 1 | 1 | 1 | 2 | 1 | 1 | 1 | 9 |
| Piazza，2013 | 1 | 1 | 1 | 1 | 2 | 1 | 1 | 1 | 9 |
| Osnabrugge,2012 | 1 | 1 | 1 | 1 | 2 | 1 | 1 | 1 | 9 |
| Kawashima,2017 | 1 | 1 | 1 | 1 | 2 | 1 | 0 | 1 | 8 |
| Sponga,2017 | 1 | 1 | 1 | 1 | 2 | 1 | 0 | 1 | 8 |
| Repossini,2017 | 1 | 1 | 1 | 1 | 2 | 1 | 0 | 1 | 8 |

**Publication bias-Funnel ploy**


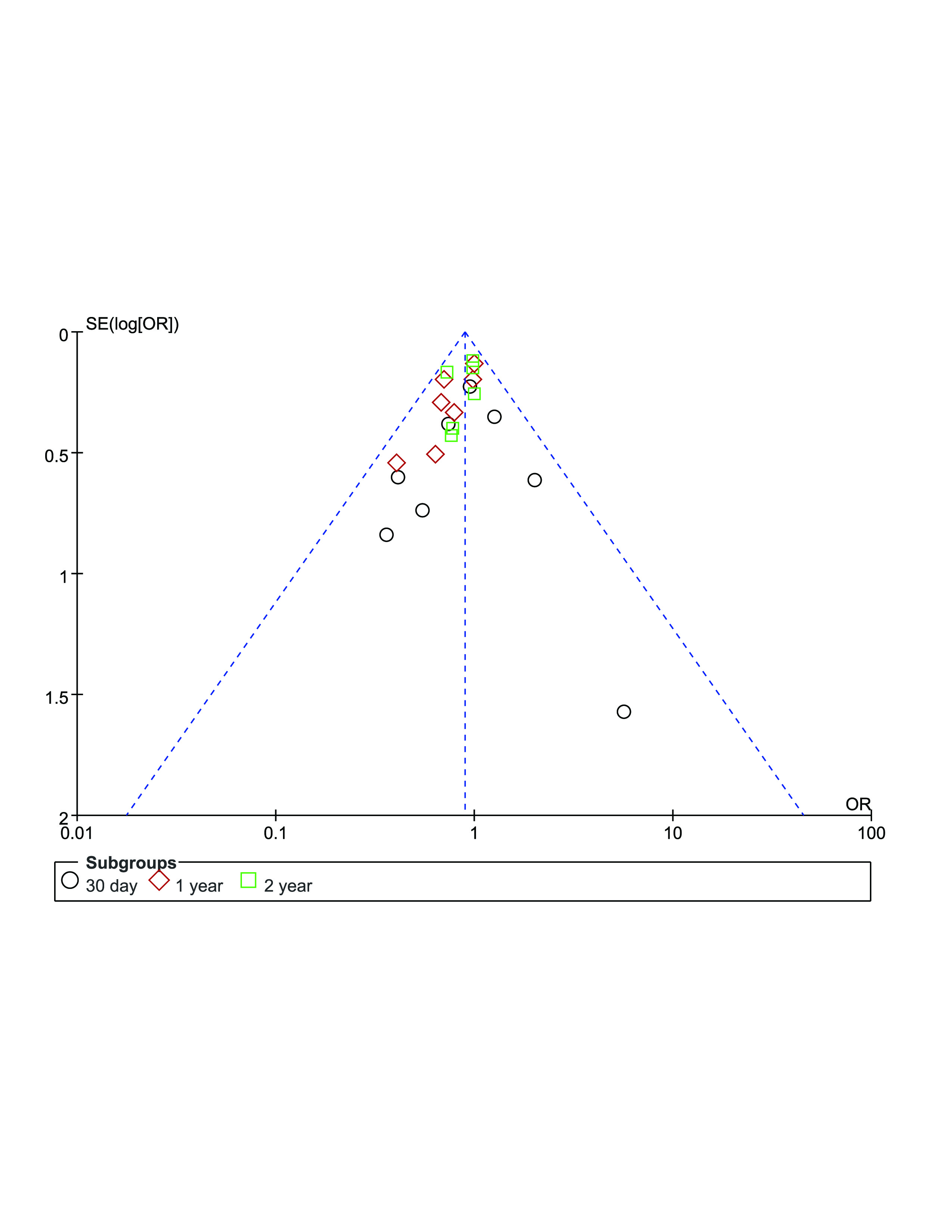

Supplement: Supplementary file 2 [file Data_Sheet_1.DOC]
