# Supplementary material for: Efficacy and Safety of Transcatheter vs. Surgical Aortic Valve Replacement in Low-to-Intermediate-Risk Patients: A Meta-Analysis
Source: Front Cardiovasc Med. 2020 Nov 16;7:590975. doi: 10.3389/fcvm.2020.590975 (PMC7701058; doi:10.3389/fcvm.2020.590975)

**Influence analysis of all-cause-death at 30-day follow-up**


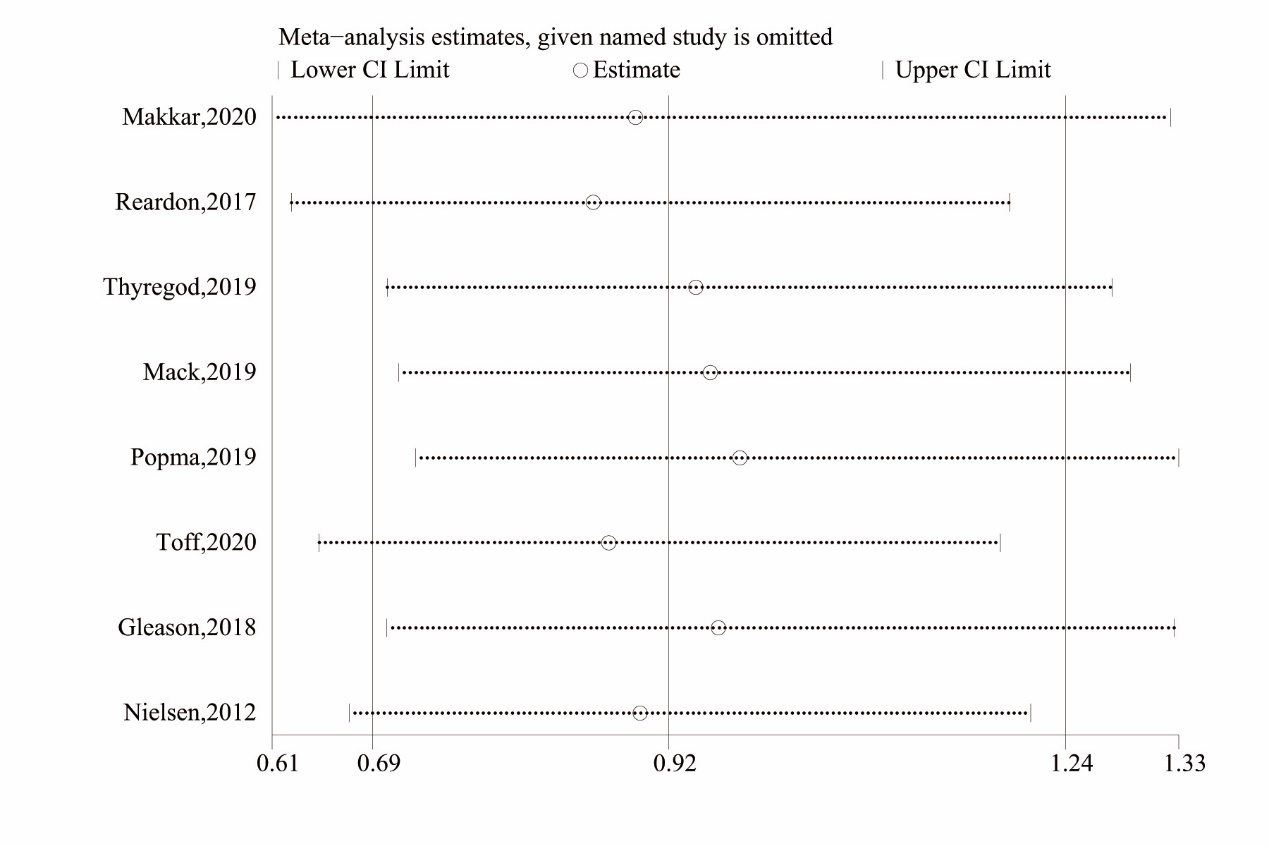


**Influence analysis of all-cause-death at 1-year follow-up**


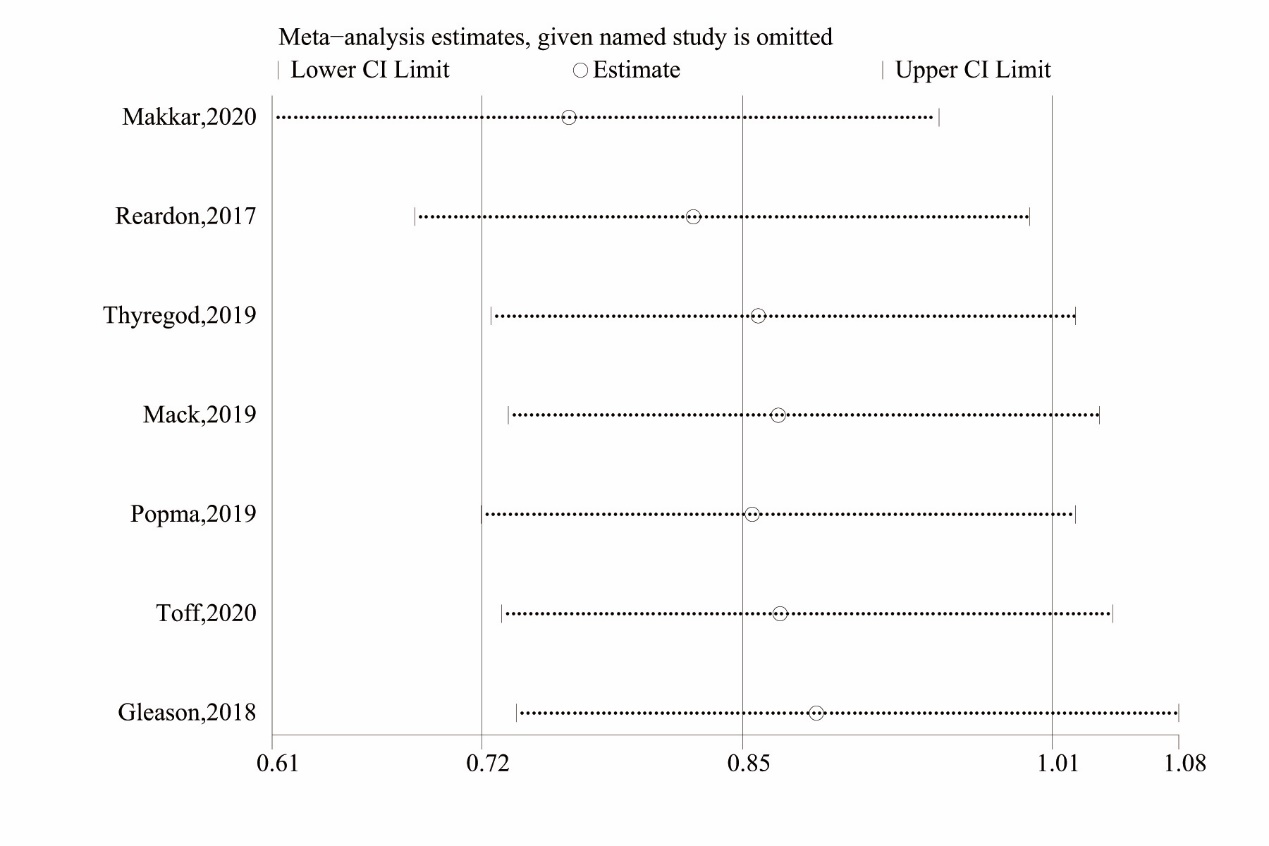


**Influence analysis of all-cause-death at 2-year follow-up**


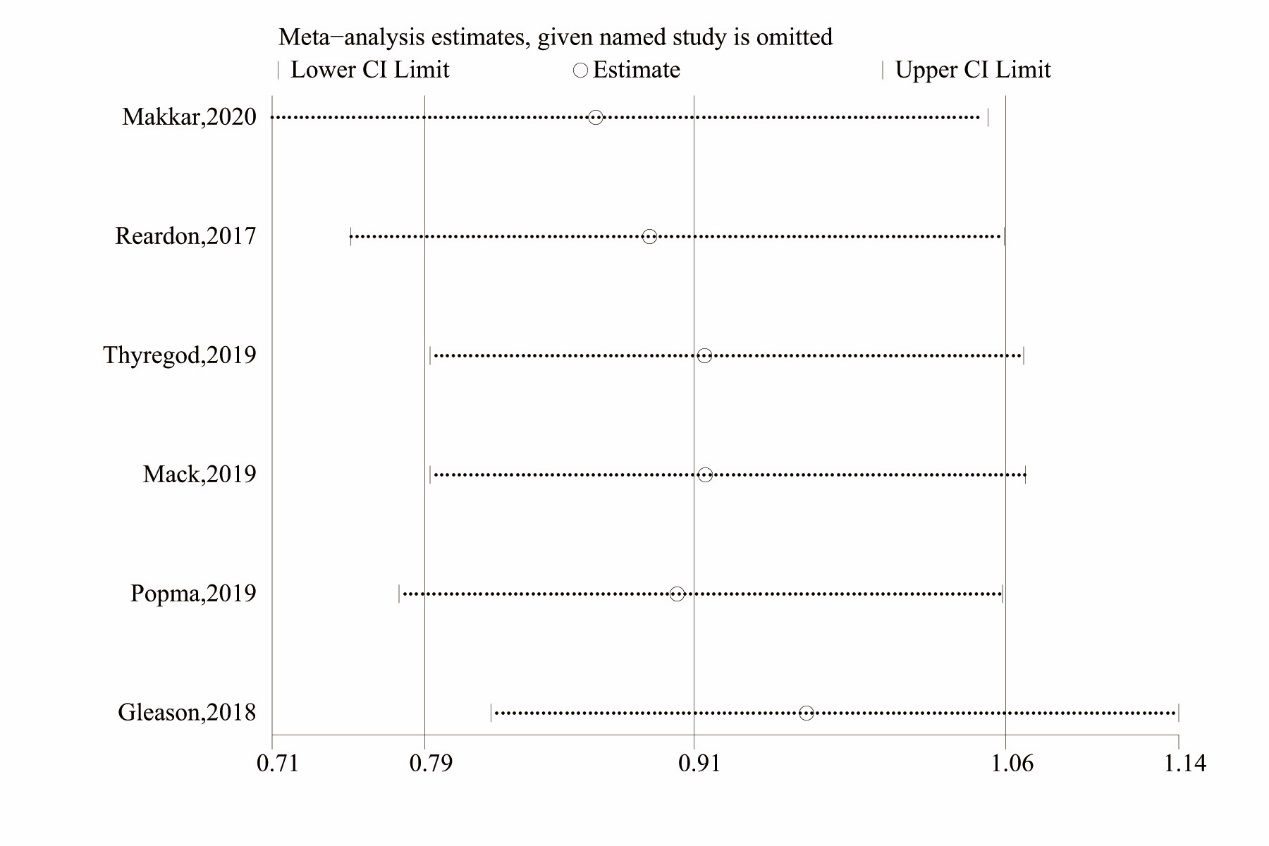

Supplement: Supplementary file 3 [file Data_Sheet_2.DOC]
